# Simulated Improvements in Influence at Work and Reduction in Sickness Absence Among Young Employees: A Nationwide Register-Based Study

**DOI:** 10.3389/ijph.2026.1609400

**Published:** 2026-04-23

**Authors:** Jeppe Karl Sørensen, Jimmi Mathisen, Jacob Pedersen, Hermann Burr, Anders Holm, Tea Lallukka, Maria Melchior, Naja Hulvej Rod, Reiner Rugulies, Børge Sivertsen, Stephen Stansfeld, Karl Bang Christensen, Ida Elisabeth Huitfeldt Madsen

**Affiliations:** 1 National Research Centre for the Working Environment, Copenhagen, Denmark; 2 Section of Epidemiology, Department of Public Health, University of Copenhagen, Copenhagen, Denmark; 3 Unit Psychosocial Factors and Mental Health, Department of Work and Health, Federal Institute for Occupational Safety and Health (BAuA), Berlin, Germany; 4 Department of Sociology, Western University, London, ON, Canada; 5 Department of Public Health, University of Helsinki, Helsinki, Finland; 6 Sorbonne Université, INSERM, Institut Pierre Louis d'Épidémiologie et de Santé Publique (IPLESP), Equipe de Recherche en Epidémiologie Sociale (ERES), Paris, France; 7 Department of Health Promotion, Norwegian Institute of Public Health, Bergen, Norway; 8 Department of Research and Innovation, Helse Fonna HF, Haugesund, Norway; 9 Centre for Psychiatry and Mental Health, Barts and the London School of Medicine, Queen Mary University of London, London, United Kingdom; 10 Section of Biostatistics, Department of Public Health, University of Copenhagen, Copenhagen, Denmark; 11 National Institute of Public Health, University of Southern Denmark, Copenhagen, Denmark

**Keywords:** influence at work, job-exposure matrix, occupational health, sickness absence, young workers

## Abstract

**Objective:**

To estimate the reduction in sickness absence associated with simulated improvements in influence at work (employees’ ability to influence how and when work tasks are performed) among young Danish employees.

**Methods:**

We used register data from the Danish Work Life Course Cohort, which included 301,185 individuals aged 15–30 who entered the labor market between 2010 and 2018 (mean follow-up: 2.6 years). Annual influence at work was assessed using a job-exposure matrix, which assigned an average level of influence based on job title. Inspired by the parametric g-formula, we used Poisson regression to predict sickness absence days under a simulated scenario in which the influence increased by one standard deviation.

**Results:**

Higher influence was associated with fewer days of sickness absence (rate ratio per one-point increase, range 1–5: 0.71, 95% CI 0.66–0.77). Simulating a standard deviation increase in influence corresponded to a reduction of 0.16 days of sickness absence per person annually, which is equivalent to an estimated reduction of 126,400 (3%) days during the follow-up period. The largest reductions were observed in care work and education.

**Conclusion:**

Simulated improvements in influence at work may lead to meaningful reductions in sickness absence among young employees.

## Introduction

In an era characterized by high employment rates and labor shortages across many occupational groups in high-income countries, the relationship between the work environment and work absenteeism has emerged as a critical area of investigation [1]. This is particularly relevant for young employees (age 15–30) at the beginning of their work life, as early increased work absenteeism may have long-term consequences [2–4]. Younger cohorts entering the labor market today are sometimes described as having different expectations regarding work, including a greater emphasis on autonomy, participation in decision-making processes, finding meaning in their work, and achieving work–life balance. However, evidence of such differences remains limited [5, 6]. In this context, psychosocial working conditions, such as influence at work, may play an important role in shaping young employees’ work environments and their risk of sickness absence. Sickness absence is one major driver of work absenteeism and is a valid predictor of health [7]. Aside from the individual perspective, the economic costs of health-related work absenteeism are substantial, with an average cost of 2.15% of the GDP across EU countries [8] and $260 billion for U.S. employers [9].

Workplace interventions have been suggested to improve health and wellbeing among employees [10]. Results from a recent umbrella review of systematic reviews covering 957 workplace intervention studies suggested that organizational interventions focusing on increasing influence at work were particularly effective in achieving favorable outcomes, including reducing sickness absence [11]. Influence at work, i.e., decision authority, refers to the degree to which a worker can influence aspects of work itself, from planning work to prioritizing tasks [12]. Low influence at work has been associated with increased sickness absence [13-17] and decreased mental well-being [16, 18] which is of great importance for young adults transitioning into the labor market [19].

Ideally, evidence supporting interventions should be derived from randomized controlled trials. However, conducting randomized trials within a work environment setting is often impossible, both ethically and in terms of feasibility [20, 21]. An alternative to trials is to analyze the potential impact of hypothetical interventions using observational data, and the parametric g-formula has been suggested [22, 23]. Recently, Mathisen et al. quantified the potential reduction in sickness absence through simulated improvements in psychosocial working conditions among middle-aged Danish hospital employees. The study compared a simulated scenario in which all individuals experienced the most desirable psychosocial work environment with the actual observed work environment [13]. However, it may be challenging to establish interventions that can improve working conditions to the most desirable level in real-life workplace settings. As current knowledge has predominantly been limited to middle-aged workers from specific sectors, it is important to investigate the potential for reducing sickness absence through improvements that can be attained more realistically in workplace settings in a nationwide cohort of younger employees. In Denmark, when employees are unable to work due to illness or injury, wage payments starting from the first day are paid by either the employer or the municipality [24]. As even small reductions in sickness absence days may benefit both individuals, workplaces, and society, addressing this question is crucial for designing targeted interventions that can contribute to the overall wellbeing of the young workforce. Thus, we aimed to investigate the following objectives: I) What is the potential predicted reduction in sickness absence days through potentially achievable improvements in influence at work among younger Danish employees, and II) to what extent do these changes vary across occupational groups?

## Methods

### Study Population

**FIGURE 1:**
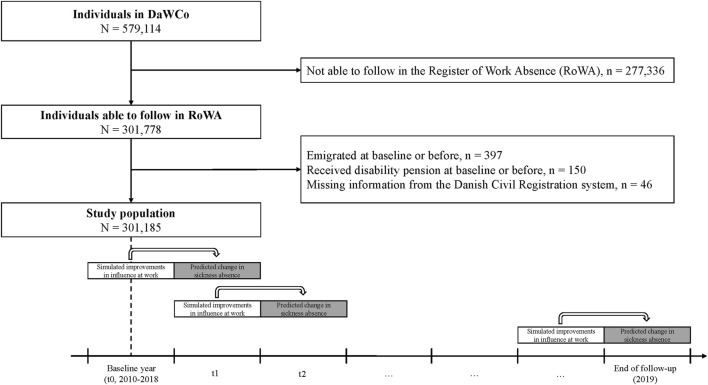
Flowchart of the study population and analytical design. The Danish Work Life Course Cohort study, Denmark, 2010–2019.

We used data from the Danish Work Life Course Cohort study (DaWCo) [25], consisting of a nationwide sample of all younger employees who entered the Danish labor market for the first time between 2010 and 2018, aged 15 and 30 (N = 579,114). The DaWCo study has been registered with and approved by the Danish Data Protection Agency under the joint notification of the National Research Centre for the Working Environment (approval no. 2015-57-0074). We followed individuals in Danish administrative registers from their entry in the labor market until the end of the follow-up period in 2019. Sickness absence data were retrieved from the Danish Register of Work Absence (RoWA). RoWA is a combination of two registers: Statistics Denmark’s “Absence and Employment” and “Periods of Absence”, and includes administrative data on daily absence for all public workplaces and a yearly sample of medium-sized and large private companies (>10 employees) [26]. Hence, we were able to follow 301,778 individuals in the RoWA database at some point during the follow-up period. We excluded individuals who emigrated (N = 397) or received a disability pension (N = 150) before or during the baseline year, and excluded 46 individuals with missing sex information from the Danish Civil Registration system. The final study population consisted of 301,185 individuals with a mean follow-up time of 2.6 years (see Figure 1). To assess the potential reduction in sickness absence across occupational groups, individuals were categorized by the Danish version of the EU’s nomenclature (NACE) into one of the following groups: knowledge, service, care, education, industrial, construction, or other [27]. The coding of occupational groups is presented in the Supplementary Table SA1.

### Sickness Absence Days

We registered the annual number of sickness absence days from 1 January 2011 to 31 December 2019 in RoWA [26]. We only included sickness absence due to one’s own sickness and disregarded absence due to work-related injuries or caring for a sick child. In Denmark, when employees are unable to work due to illness or injury, it is typically the employer’s responsibility to provide wage payments starting from the first day of absence. After 30 days of absence, employers are reimbursed for sickness benefits by the municipality [24].

### Influence at Work

We estimated the yearly occupational level of influence at work using a job-exposure matrix (JEM) based on survey data from 17,591 respondents (aged 18–64) from the 2012 Work Environment and Health in Denmark study (WEHD) [28]. In the WEHD, the individual level of influence at work was measured using two items: “How often can you influence how you solve your work tasks?” and “How often can you influence when you solve your work tasks?” with response options being “Never”, “Seldom”, “Sometimes”, “Often”, and “Always”. The JEM was estimated as the predicted mean level of influence at work, ranging from 1 to 5, with higher scores indicating a higher level of influence. The mean level was estimated separately for women and men, using a linear mixed model that modeled the effect of age using splines to account for non-linear effects. A random intercept for job title (Danish version of the International Standard Classification of Occupations, DISCO-08) was included to produce job title-, sex-, and age-specific JEMs. The sex-specific Interclass Correlation Coefficient (ICC) was 0.07 and 0.09 for women and men, respectively [28]. The estimated JEM level of influence at work was assigned as a time-varying exposure to each individual in DaWCo based on their yearly job title, sex, and age. In line with previous studies, the 18-year-old-specific JEM was assigned to employees aged 15 to 17 [15].

### Other Covariates

Information on sociodemographic characteristics, socioeconomic status, occupation, and health was included from Danish national administrative registers [29, 30]. Sociodemographic covariates included sex (women or men), age, civil status (single or living alone, or married or in a registered partnership), and ethnicity (born in Denmark with no migration background, born outside Denmark, or born in Denmark with both immigrant parents). Socioeconomic status was measured by annual disposable income after taxes and occupational grade (professional, intermediate, or routine). Additionally, we included information on childhood socioeconomic status, measured by the highest educational attainment of the mother and father and the participant’s current labor market affiliation when they were 15 years old. We measured health as the annual number of health services used within primary healthcare (e.g., general practitioners, physiotherapists, or chiropractors) 1 year before the JEM assignments and hospital diagnoses of somatic diseases (type 2 diabetes, coronary heart disease, stroke, cancer, asthma, and chronic obstructive pulmonary disease, the World Health Organization’s priority non-communicable chronic diseases target for prevention) [31] and mental disorders (ICD-10 psychiatric diagnoses) before labor market entry. Furthermore, we included a JEM on physical workload, as physical workload has been found to be an important confounder strongly related to sickness absence [32]. We adjusted for the number of sickness absence days in the previous year because a history of sickness absence have been associated with both subsequent sickness absence and current employment [2-4], and may therefore influence the assignment of JEM scores. Finally, to account for the design of the cohort, we included calendar year, year of labor market entry, years since labor market entry, years employed, and employment sector (public or private). Covariates were defined as time-varying, except for sex, migration background, and somatic diseases and mental disorders before labor market entry, which were defined as time-invariant.

### Analytical Framework

We estimated the predicted changes in annual sickness absence days under a simulated exposure scenario inspired by the parametric g-formula [23, 33]. The parametric g-formula is a method used to estimate causal effects in settings with time-varying exposures and confounders, such as in occupational epidemiology. It simulates outcomes under hypothetical interventions by modeling the joint distribution of exposures, covariates, and outcomes over time. [22, 23, 33]. The approach relies on simulated exposure contrasts, either predicting the risk of a specific health outcome as an etiological effect, corresponding to effect estimates from randomized controlled trials, or a cohort effect, corresponding to a scenario where all individuals in a cohort are simulated to have the most desirable level of the exposure [22]. However, this approach may not fully align with exposure in the psychosocial work environment, as it may be challenging or even impossible to implement real-life interventions that can improve working conditions to the most desirable level. Additionally, there is no consensus in the literature on what constitutes potentially achievable improvements in influence among younger employees, and these improvements may vary across different occupational contexts. Therefore, we present the predicted change in sickness absence days under the hypothetical scenario in which each individual was simulated to have a one standard deviation higher level of influence at work. For each job title, sex, and age-specific JEM on influence at work, we used the standard deviation derived from the linear mixed model used to estimate the JEM [28]. Hence, the simulated improvements in influence at work varied across job titles, with absolute increases between 0.06 and 0.16 on a scale ranging from 1 to 5, reflecting the observed variance in influence at work by job title. The average level of observed influence and the corresponding simulated improved influence across job titles can be found in Supplementary Table S2.

We compared the predicted annual sickness absence days in the hypothetical exposure scenarios with the predicted annual sickness absence days in the observed scenario. Annual sickness absence days were estimated using a multi-level Poisson regression model adjusted for sex, age, years since labor market entry, years employed, calendar year, civil status, ethnicity, disposable income, employment sector, occupational grade, health service use, somatic diseases and mental disorders before labor market entry, physical workload, childhood socioeconomic status, and previous sickness absence. The level of influence at work was associated with the annual number of sickness absence days the following year to ensure a longitudinal design (see Figure 1). The logarithm of the total number of days during follow-up was used as an offset to accommodate unequal days at risk, and a scale parameter was included to address overdispersion. We included a multilevel approach to account for multiple observations at the individual level and clustering of working conditions at the job title level. Periods of non-employment during the follow-up period were considered time not at risk, and individuals were censored on the date of emigration, receipt of a disability pension, death, or the end of the follow-up period on 31 December 2019, whichever came first. To quantify the uncertainty of the estimated potential reduction in sickness absence, we estimated 95% confidence intervals (CI) using bootstrapping methods. All analyses were conducted for all individuals and separately for occupational groups. Sensitivity analyses were conducted separately for women and men and without adjustment for the occupationally-assessed physical workload.

All analyses were conducted using SAS 9.4.

## Results

Table 1 shows the baseline characteristics of the 301,185 individuals included at their labor market entry, and Supplementary Table S2 shows the baseline characteristics across occupational groups. Briefly, the cohort consisted of 160,104 women (53.2%) and 141,081 men (46.8%), with a mean age of 20.1 (3.9 SD). The majority of the individuals were employed in service work (61.4%) at baseline. During follow-up, a larger proportion of women became employed in care work and a larger proportion of men in knowledge work (Supplementary Figure S1). Supplementary Table S2, presents baseline characteristics across occupational groups.

**TABLE 1:** Baseline characteristics of young employees entering the labor market. The Danish Work Life Course Cohort study, Denmark, 2010–2019.

Baseline characteristics	n (%)
Total	301,185
Sex	
Women	160,104 (53.2%)
Men	141,081 (46.8%)
Age in years. Mean (SD)	20.1 (3.9)
15–19	164,816 (54.7%)
20–25	100,873 (33.5%)
>25	35,496 (11.8%)
Civil status	
Single or living alone	85,550 (28.4%)
Married or in a registered partnership	183,213 (60.8%)
Unknown	32,422 (10.8%)
Ethnicity	
Born in Denmark with no migration background	223,397 (74.2%)
Born outside Denmark	57,846 (19.2%)
Born in Denmark with both immigrant parents	19,942 (6.6%)
Annual disposable income (EUR). Mean (SD)	9,064 (8,547.1)
<10,000 EUR	201,574 (66.9%)
10,000–20,000 EUR	72,413 (24.0%)
20,000–30,000 EUR	18,464 (6.1%)
30,000–40,000 EUR	6,105 (2.0%)
>40,000 EUR	2,395 (0.8%)
Employment sector	
Public	117,891 (39.1%)
Private	183,294 (60.9%)
Occupational level	
Professional	27,743 (9.2%)
Semi-professional and clerical	158,853 (52.7%)
Routine	114,440 (38.0%)
Somatic diseases	
No	280,730 (93.2%)
Yes	20,455 (6.8%)
Mental disorders	
No	279,380 (92.8)
Yes	21,805 (7.2)
Annual health service use. Mean (SD)	11.4 (14.2)
0	71,036 (23.6)
1–3	61,796 (20.5)
4–7	58,904 (19.6)
8–15	58,280 (19.4)
16+	51,157 (17.0)
Annual sickness absence	
Mean days (SD)	3.2 (11.9)

**FIGURE 2:**
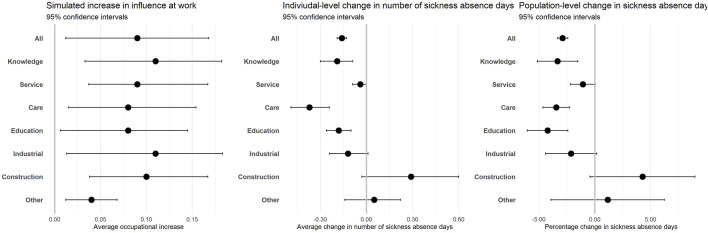
Predicted average individual-level and population-level changes in sickness absence days associated with simulated increase in influence at work among young employees. The Danish Work Life Course Cohort study, Denmark, 2010–2019.

Table 2 presents the estimated rate ratios (RR) and 95% confidence intervals (CI) for the association between a one-point increase in the level of influence at work and the number of annual sickness absence days. During 788,527 person-years, we recorded 4,392,272 sickness absence days, corresponding to 5.6 sickness absence days per person-year. In model 1, including sex, age, years since labor market entry, years employed, calendar year, and employment sector, we found that a one-point higher level of influence at work at the occupational level was associated with fewer sickness absence days with an RR of 0.33 (95% CI: 0.30–0.35). After additional adjustment for socioeconomic status, health, and previous sickness absence, the association weakened, and a one-point higher level of influence was associated with sickness absence days with an RR of 0.71 (95% CI: 0.66–0.77). The change from model 1 to the fully adjusted model was mainly driven by the adjustment for previous sickness absence days (Supplementary Table S2). Figure 2 presents the estimated predicted change in annual sickness absence days. Simulated increases in influence at work by one standard deviation were associated with an individual-level reduction of 0.16 (95% CI: 0.13–0.19) annual sickness absence days and a total population-level reduction of 126,400 (95% CI: 105,885–146,914) sickness absence days. The total population-level reduction corresponded to a 2.9% (3.3%–2.4%) reduction in the total number of sickness absence days during follow-up between the observed and simulated scenarios.

**TABLE 2:** The association between inlfluence at work and annual sickness absence days among young employees, presented as rate ratio with 95% confidence intervals. The Danish Work Life Course Cohort study, Denmark, 2010–2019.

Baseline characteristics	Person years	Sickness absence days	Sickness absence days per person years	One point higher influence at work	
				Model 1 RR (95% CI)	Model 2 RR (95% CI)
All	788,527	4,392,272	5.6	0.33 (0.30–0.35)	0.71 (0.66–0.77)
Knowledge	78,813	447,119	5.7	0.51 (0.42–0.62)	0.71 (0.56–0.90)
Service	405,884	1,626,709	4.0	0.56 (0.49–0.64)	0.88 (0.77–1.02)
Care	147,464	1,571,616	10.7	0.57 (0.48–0.67)	0.62 (0.52–0.75)
Education	59,228	251,000	4.2	0.11 (0.08–0.17)	0.57 (0.37–0.89)
Industrial	53,012	294,209	5.5	0.36 (0.31–0.41)	0.84 (0.70–1.01)
Construction	13,746	92,196	6.7	0.93 (0.41–2.15)	1.49 (0.63–3.52)
Other	30,379	109,423	3.6	0.16 (0.08–0.31)	1.30 (0.46–3.69)

RR, Rate ratio; CI, Confidence intervals. Model 1 adjustment for sex, age, years since labor market entry, years employed, and calendar year. Model 2 further adjusted for civil status, ethnicity, income, employment sector, occupational grade, health service use, somatic diseases, and mental disorders before labor market entry, physical workload, childhood socioeconomic status, and previous sickness absence.

Across occupational groups, influence at work was statistically significantly associated with sickness absence in knowledge work (0.71, 95% CI: 0.56–0.90), care work (0.62, 95% CI: 0.52–0.75), and education (0.57, 95% CI: 0.37–0.89). In service work (0.88, 95% CI: 0.77–1.02), industrial work (0.84, 95%CI: 0.70–1.01), construction work (1.49, 95% CI: 0.63–3.52) and other jobs (1.30, 95% CI: 0.46–3.69) influence at work was not statistically associated with sickness absence days (Table 2). The largest individual-level reduction in annual sickness absence days was found in care work (individual-level change: 0.37 days, 95% CI: 0.24–0.49) and the largest population-level change in sickness absence days was found in education (population-level change: 4.4%, 95% CI: 2.4%–6.0%) (Figure 2, Supplementary Tables S3, S4).

Supplementary analyses showed similar individual-level changes in the number of sickness absence days among women and men, but greater population-level changes in sickness absence days among women (Supplementary Table S5). Analyses without adjustment for physical workload indicated that adjustment for physical workload in the fully adjusted model attenuated the association and changed the direction in construction work (RR from 1.49 to 0.91) and other jobs (RR from 1.30 to 0.60); however, the confidence intervals included unity (Supplementary Table S2).

## Discussion

In this large nationwide register-based cohort of 301,185 young employees who were followed from their first entry into the labor market between 2010 and 2018, we identified an association between influence at work and sickness absence days. Simulated improvements in influence at work with one standard deviation, corresponding to an average of 2% increase or 0.09 on a scale from 1 to 5, were associated with a small individual-level reduction in sickness absence days, which corresponded to a total predicted reduction of 126,400 sickness absence days at the population level during the follow-up period. We found similar associations across the majority of occupational groups, with the largest potential for reducing sickness absence days in care work and education.

Our findings are in line with previous research on the association between influence at work and sickness absence [13–15]. However, the majority of these studies have established an association among middle-aged employees, and only a few have reported similar associations among young employees [14], including one study based on the same cohort [15]. As an example, in a study of 56,867 Swedish employees, Wang et al. reported that low job control was associated with a lower risk of long-term sickness absence (31─365 days) among young employees aged 26─35. However, differences in the defined sickness absence outcomes and adjustments for socioeconomic status between the studies make direct comparison of effect estimates difficult.

We found that 126,400 sickness absence days could theoretically be prevented through potential achievable improvements in influence at work. Since few studies have investigated occupational-level interventions on influence at work among younger employees, it can be questioned whether the simulated improvements could be effectively translated into real-world settings. However, existing research does offer some insight. For instance, previous organizational-level interventions have shown a correlation between increased influence at work and reduced sickness absence [34]. One notable study conducted in the United Kingdom involving 97 office workers employed a participatory intervention aimed at restructuring work to enhance employee discretion and work autonomy. After a year of follow-up, the intervention group experienced, on average, a 23% increase in job control [35]. Another study, involving 264 manual workers in the Netherlands, implemented an intervention combining psychosocial skills training and health promotion programs, resulting in a 9% increase in job control after 3 years [36]. While these studies showed promising results, it is important to note that the participants were predominantly middle-aged employees, raising questions about the generalizability of these findings to younger employees. Consequently, there remains a need for further investigation into the effectiveness of interventions targeting influence at work among younger employees.

To the best of our knowledge, no previous study has predicted a reduction in sickness absence days through simulated improvements in influence at work among younger adults. Nevertheless, the magnitude of the potential reduction in sickness absence days reported in this study aligns with previous findings from two Danish studies. These studies indicated that between 4% and 10% of sickness absence could potentially be prevented by improving influence at work to the most desirable level [13, 37]. However, both studies were conducted on middle-aged employees in specific occupational settings, and only the study by Mathisen et al. simulated improvements in influence at work [13].

The strengths of the study include its large, nationwide cohort of 301,185 young employees in Denmark who entered the labor market between 2010 and 2018. Combining this register-based cohort with an annual JEM on influence at work and detailed information on sickness absence enabled us to establish associations for all young employees and conduct occupational group-specific analyses. We applied the parametric g-formula to estimate the associations of a potentially achievable intervention, which allowed us to provide both simulated individual-level and population-level reduction in sickness absence days, which, in general, are more informative for decision-makers and public health practitioners than standard regression analyses [23].

In our statistical analyses, we focused on only one work environment factor. Different factors within the psychosocial working environment have been reported to be associated with sickness absence [16]; however, in this study, we specifically focused on influence at work (i.e., decision authority) for the following reasons. First, organizational interventions aimed at increasing influence at work have been found to be effective in improving various favorable outcomes, including sickness absence [10, 11]. Second, we previously found that influence at work, as measured at the occupational level, is associated with sickness absence spells among younger women and men and across the majority of occupational groups [15]. As many working conditions within the psychosocial work environment often overlap [12], it is possible that interventions targeting influence at work may also impact other aspects of the work environment and hence affect sickness absence differently. In this study, we included income and occupational grade as measures of socioeconomic status and additionally included information on childhood socioeconomic status and physical workload as proxy measures. Considering that influence at work is so closely linked with socioeconomic status and that socioeconomic status is also associated with health, as health-hazardous behaviors are more common among individuals of low socioeconomic status [38], concerns about residual confounding by socioeconomic status are probable. We did not adjust the analyses for education because we expect substantial over-adjustment, as the JEM on influence is based on job title, which is highly correlated with education. Additionally, the JEM on influence at work was based on an unvalidated questionnaire, and the ICC values indicated moderate reliability. However, the predicted variability of the JEM has been shown in a previous study to be acceptable in relation to sickness absence [15]. Another limitation of the JEM is the misclassification of exposure, as individual employees working in jobs with, on average, a low level of influence at work are not necessarily actually exposed to low influence. Since sickness absence days are measured independently of exposure, we expect the misclassification to be non-differential, which potentially could lead to an underestimation of the predicted change in sickness absence. Hence, the magnitude of the potential reduction in sickness absence should be interpreted with the understanding that the potential for reducing sickness absence could be greater. Moreover, we included information on sickness absence for all public employees and a yearly sample of private employees from companies with 10 or more employees. Based on an estimation from the Danish Agency for Labour Market and Recruitment, conducted for the purpose of this study, approximately 26% of all younger employees between 15 and 30 years old were employed in small companies with fewer than 10 employees in 2019. Hence, generalizability to private employees from small companies may be limited. Another limitation is that our register-based data did not include information on individual work-related attitudes or expectations. Differences in work-related expectations among cohorts entering the labor market may influence sickness absence behavior; however, these differences could not be examined in the present study. Finally, information on the length of sickness absence was only available when individuals were employed, which could cause some misclassification of longer sickness absence spells.

The generalizability of the findings should be considered in light of the Danish labor market and welfare context. Denmark has relatively comprehensive sickness benefit regulations (e.g., providing paid salary from the first day of absence), which may differ from systems in other countries. Therefore, the magnitude of the predicted reductions in sickness absence may not directly translate to other national contexts. However, the overall relationship between influence at work and sickness absence is likely to be relevant in similar labor market settings.

In conclusion, this study found that influence at work was an important predictor of sickness absence among younger employees at the beginning of their working lives. Based on simulated scenarios, we found that potentially achievable interventions aimed at improving influence at work could have a small but significant effect on reducing the number of sickness absence days, especially among employees working in care and education. These findings highlight the critical role of addressing psychosocial working conditions, including influence at work, to reduce sickness absence days among younger employees, emphasizing the need for implementing interventions and conducting further research to optimize workforce wellbeing. Importantly, these results offer decision-makers a clear evidence base to prioritize interventions that enhance influence at work. Improving young workers’ opportunities to participate in decisions and exercise autonomy may not only support better health and work engagement but also contribute to reducing the substantial individual and economic burdens associated with sickness absence. Targeted initiatives in occupations with the greatest potential for improvement, particularly in care work and education, may therefore represent an efficient strategy for strengthening workforce sustainability. Continued research is needed to assess the real-world effectiveness of these interventions among young employees and to inform how organizations can best implement and sustain improvements in influence at work.

## Data Availability

The data analysed in this study were obtained from Statistics Denmark. Due to Danish data protection regulations, the data are not publicly available. Access can be granted to authorized researchers following application to Statistics Denmark and approval by the National Research Centre for the Working Environment. For further information, please contact the corresponding author (jks@nfa.dk) or the National Research Centre for the Working Environment (nfa@nfa.dk).
