# Supplementary material for: Simulated Improvements in Influence at Work and Reduction in Sickness Absence Among Young Employees: A Nationwide Register-Based Study
Source: Int J Public Health. 2026 Apr 23;71:1609400. doi: 10.3389/ijph.2026.1609400 (PMC13149206; doi:10.3389/ijph.2026.1609400)

**Supplementary material**

**Simulated Improvements in Influence at Work and Reduction in Sickness Absence Among Young Employees: A Nationwide Regiser-Based Study**

[Appendix 1. Coding of occupation, occupational group and simulated improvements in influence at work 2](#_Toc215489127)

[Appendix 2. Supplementary findings 7](#_Toc215489128)

## Appendix 1. Coding of occupation, occupational group and simulated improvements in influence at work

We used the Dansk Branchekode 2007 (DB07), which is the Danish version of EU’s nomenclature (NACE) to classify individuals into occupational group (knowledge, service, care, education, industrial, construction, and other). Coding of occupational grade and occupational group is presented in the table S1.

**Table S1.** Coding of occupation and occupational group

| Occupational grade | | | | | | | | |
| --- | --- | --- | --- | --- | --- | --- | --- | --- |
| **Professional** | | **Semi-professional and clerical** | | | | **Routine** | | |
| Managers  Professionals | | Technicians and associate professionals  Clerical support workers  Services and sales worker | | | | Skilled agricultural, forestry, and fishery workers  Craft and related trades workers  Plant and machine operators, and assemblers  Elementary occupations  Armed forces occupations | | |
| **Occupational group** | | | | | | | | |
| **Knowledge** | **Service** | | **Care** | **Education** | **Industrial** | | **Construction** | **Other** |
| Information and communication  Financial and insurance activities    Real estate activities  Professional, scientific and technical activities  Public administration and defense compulsory social security | Whole sale and retail trade; repair of motor vehicles and motorcycles  Transport and storage  Accommodation and food service activities  Arts, entertainment and recreation  Administrative and support service activity  Other service activities | | Human health and social work | Education | Agriculture, forestry and fishing  Mining and quarrying  Manufacturing  Electricity, gas, steam and air conditioning supply  Water supply; sewerage, waste management and remediation activities | | Construction | Activities of household as employers  Activities of extraterritorial organizations and bodies  Unknown |

**Table S2.** Average observed level of influence at work (standard deviation (SD)) and simulated improved influence at work across occupational title (DISCO-08, level 3).

| **DISCO-08 (level 3)** | **Occupational title** | **Observed influence at work (SD)** | **Simulated improved  influence at work** |
| --- | --- | --- | --- |
| 122 | Sales, Marketing and Development Managers | 4.32 (0.12) | 4.44 |
| 231 | University and Higher Education Teachers | 4.31 (0.08) | 4.38 |
| 133 | Information and Communications Technology Service managers | 4.30 (0.13) | 4.43 |
| 121 | Business Services and Administration Managers | 4.26 (0.13) | 4.39 |
| 132 | Manufacturing, Mining, Construction and Distribution Managers | 4.23 (0.14) | 4.37 |
| 242 | Administration Professionals | 4.22 (0.14) | 4.35 |
| 143 | Other Services Managers | 4.21 (0.14) | 4.35 |
| 260 | Legal, Social and Cultural Professionals | 4.21 (0.11) | 4.32 |
| 314 | Life Science Technicians and Related Associate Professionals | 4.21 (0.14) | 4.34 |
| 214 | Engineering Professionals (excluding Electrotechnology) | 4.20 (0.11) | 4.32 |
| 216 | Architects, Planners, Surveyors and Designers | 4.20 (0.14) | 4.33 |
| 211 | Physical and Earth Science Professionals | 4.19 (0.13) | 4.33 |
| 210 | Non-commissioned Armed Forces Officers | 4.19 (0.13) | 4.32 |
| 134 | Professional Services Managers | 4.18 (0.15) | 4.33 |
| 343 | Artistic, Cultural and Culinary Associate Professionals | 4.18 (0.14) | 4.32 |
| 142 | Retail and Wholesale Trade Managers | 4.18 (0.14) | 4.32 |
| 243 | Sales, Marketing and Public Relations Professionals | 4.18 (0.14) | 4.32 |
| 212 | Mathematicians, Actuaries and Statisticians | 4.18 (0.13) | 4.31 |
| 141 | Hotel and Restaurant Managers | 4.18 (0.14) | 4.32 |
| 213 | Life Science Professionals | 4.18 (0.15) | 4.33 |
| 215 | Electrotechnology Engineers | 4.17 (0.13) | 4.30 |
| 412 | Secretaries (general) | 4.16 (0.10) | 4.26 |
| 111 | Legislators and Senior Officials | 4.16 (0.14) | 4.30 |
| 330 | Business and Administration Associate Professionals | 4.16 (0.13) | 4.29 |
| 226 | Other Health Professionals | 4.16 (0.11) | 4.28 |
| 230 | Teaching Professionals | 4.16 (0.13) | 4.29 |
| 241 | Finance Professionals | 4.15 (0.12) | 4.28 |
| 200 | Professionals | 4.15 (0.10) | 4.25 |
| 265 | Creative and Performing Artists | 4.14 (0.15) | 4.30 |
| 342 | Sports and Fitness Workers | 4.14 (0.15) | 4.29 |
| 410 | General and Keyboard Clerks | 4.14 (0.10) | 4.24 |
| 240 | Business and Administration Professionals | 4.14 (0.14) | 4.27 |
| 315 | Ship and Aircraft Controllers and Technicians | 4.13 (0.12) | 4.26 |
| 341 | Legal, Social and Religious Associate Professionals | 4.13 (0.13) | 4.26 |
| 112 | Managing Directors and Chief Executives | 4.13 (0.12) | 4.25 |
| 251 | Software and Applications Developers and Analysts | 4.13 (0.12) | 4.25 |
| 811 | Mining and Mineral Processing Plant Operators | 4.13 (0.16) | 4.29 |
| 332 | Sales and Purchasing Agents and Brokers | 4.12 (0.09) | 4.22 |
| 300 | Building and Related Trades Workers (excluding Electricians) | 4.12 (0.16) | 4.27 |
| 263 | Social and Religious Professionals | 4.11 (0.13) | 4.24 |
| 413 | Keyboard Operators | 4.11 (0.11) | 4.22 |
| 220 | Health Professionals | 4.11 (0.13) | 4.24 |
| 411 | General Office Clerks | 4.11 (0.06) | 4.16 |
| 262 | Librarians, Archivists and Curators | 4.11 (0.12) | 4.23 |
| 333 | Business Services Agents | 4.11 (0.13) | 4.24 |
| 331 | Financial and Mathematical Associate Professionals | 4.09 (0.10) | 4.19 |
| 225 | Veterinarians | 4.09 (0.11) | 4.20 |
| 233 | Secondary Education Teachers | 4.08 (0.09) | 4.18 |
| 400 | Clerical Support Workers | 4.08 (0.09) | 4.17 |
| 611 | Market Gardeners and Crop Growers | 4.07 (0.16) | 4.23 |
| 514 | Hairdressers, Beauticians and Related Workers | 4.06 (0.12) | 4.18 |
| 351 | Information and Communications Technology Operations and User Support Technicians | 4.06 (0.14) | 4.20 |
| 621 | Forestry and Related Workers | 4.06 (0.16) | 4.22 |
| 311 | Physical and Engineering Science Technicians | 4.06 (0.13) | 4.18 |
| 235 | Other Teaching Professionals | 4.05 (0.11) | 4.17 |
| 261 | Legal Professionals | 4.05 (0.13) | 4.18 |
| 622 | Fishery Workers, Hunters and Trappers | 4.05 (0.16) | 4.21 |
| 612 | Animal Producers | 4.05 (0.16) | 4.20 |
| 021 | Non-commissioned Armed Forces Officers | 4.05 (0.12) | 4.16 |
| 232 | Vocational education teachers | 4.04 (0.13) | 4.18 |
| 600 | Skilled Agricultural, Forestry and Fishery Workers | 4.04 (0.16) | 4.20 |
| 325 | Other Health Associate Professionals | 4.04 (0.12) | 4.17 |
| 313 | Process Control Technicians | 4.04 (0.14) | 4.18 |
| 613 | Mixed Crop and Animal Producers | 4.03 (0.16) | 4.19 |
| 754 | Other Craft and Related Workers | 4.03 (0.14) | 4.17 |
| 234 | Primary School and Early Childhood Teachers | 4.02 (0.07) | 4.10 |
| 741 | Electrical Equipment Installers and Repairers | 4.02 (0.08) | 4.10 |
| 252 | Database and Network Professionals | 4.02 (0.14) | 4.16 |
| 817 | Wood Processing and Papermaking Plant Operators | 4.02 (0.14) | 4.16 |
| 512 | Cooks | 4.01 (0.14) | 4.15 |
| 264 | Authors, Journalists and Linguists | 4.01 (0.12) | 4.12 |
| 011 | Commissioned Armed Forces Officers | 4.01 (0.11) | 4.12 |
| 752 | Wood Treaters, Cabinet-makers and Related Trades Workers | 4.01 (0.16) | 4.16 |
| 352 | Telecommunications and Broadcasting Technicians | 4.01 (0.16) | 4.17 |
| 322 | Nursing and Midwifery Associate Professionals | 4.00 (0.14) | 4.14 |
| 334 | Administrative and Specialized Secretaries | 4.00 (0.09) | 4.09 |
| 818 | Other Stationary Plant and Machine Operators | 3.99 (0.15) | 4.15 |
| 522 | Shop Aalespersons | 3.99 (0.06) | 4.05 |
| 832 | Car, Van and Motorcycle Drivers | 3.99 (0.14) | 4.13 |
| 431 | Numerical Clerks | 3.99 (0.13) | 4.12 |
| 700 | Craft and Related Trades Workers | 3.99 (0.09) | 4.08 |
| 432 | Material Recording and Transport Clerks | 3.99 (0.12) | 4.11 |
| 712 | Building Finishers and Related Trades Workers | 3.98 (0.11) | 4.10 |
| 521 | Street and Market Salespersons | 3.98 (0.12) | 4.10 |
| 812 | Metal Processing and Finishing Plant Operators | 3.98 (0.13) | 4.11 |
| 710 | Building and Related Trades Workers (excluding Electricians) | 3.97 (0.15) | 4.12 |
| 711 | Building Frame and Related Trades Workers | 3.96 (0.09) | 4.06 |
| 931 | Mining and Construction Labourers | 3.96 (0.13) | 4.09 |
| 510 | Personal Service Workers | 3.96 (0.13) | 4.09 |
| 713 | Painters, Building Structure Cleaners and Related Trades Workers | 3.96 (0.14) | 4.10 |
| 324 | Veterinary Technicians and Assistants | 3.96 (0.14) | 4.09 |
| 722 | Blacksmiths, Toolmakers and Related Trades Workers | 3.95 (0.10) | 4.05 |
| 911 | Domestic, Hotel and Office Cleaners and Helpers | 3.95 (0.11) | 4.05 |
| 932 | Manufacturing Labourers | 3.94 (0.12) | 4.06 |
| 335 | Government Regulatory Associate Professionals | 3.94 (0.14) | 4.08 |
| 941 | Food Preparation Assistants | 3.94 (0.13) | 4.07 |
| 723 | Machinery Mechanics and Repairers | 3.93 (0.11) | 4.04 |
| 952 | Street Vendors (excluding Food) | 3.93 (0.14) | 4.07 |
| 900 | Elementary Occupations | 3.93 (0.14) | 4.07 |
| 720 | Metal, Machinery and Related Trades Workers | 3.93 (0.12) | 4.05 |
| 222 | Nursing and Midwifery Professionals | 3.93 (0.07) | 3.99 |
| 921 | Agricultural, Forestry and Fishery Labourers | 3.93 (0.14) | 4.06 |
| 513 | Waiters and Bartenders | 3.92 (0.14) | 4.06 |
| 515 | Building and Housekeeping Supervisors | 3.92 (0.11) | 4.02 |
| 753 | Garment and Related Trades Workers | 3.91 (0.14) | 4.05 |
| 961 | Refuse Workers | 3.91 (0.16) | 4.07 |
| 524 | Other Sales Workers | 3.91 (0.14) | 4.05 |
| 933 | Transport and Storage Labourers | 3.91 (0.11) | 4.01 |
| 951 | Street and Related Service Workers | 3.90 (0.14) | 4.04 |
| 523 | Cashiers and Ticket Clerks | 3.90 (0.11) | 4.01 |
| 321 | Medical and Pharmaceutical Technicians | 3.89 (0.12) | 4.02 |
| 912 | Vehicle, Window, Laundry and Other Hand Cleaning Workers | 3.89 (0.14) | 4.03 |
| 721 | Sheet and Structural Metal Workers, Moulders and Welders, and Related Workers | 3.88 (0.13) | 4.02 |
| 532 | Personal Care Workers in Health Services | 3.88 (0.07) | 3.95 |
| 815 | Textile, Fur and Leather Products Machine Operators | 3.88 (0.15) | 4.03 |
| 531 | Child Care Workers and Teachers' Aides | 3.87 (0.08) | 3.95 |
| 421 | Tellers, Money Collectors and Related Clerks | 3.85 (0.15) | 4.00 |
| 511 | Travel Attendants, Conductors and Guides | 3.83 (0.14) | 3.98 |
| 751 | Food Processing and Related Trades Workers | 3.83 (0.14) | 3.98 |
| 821 | Assemblers | 3.82 (0.13) | 3.95 |
| 813 | Chemical and Photographic Products Plant and Machine Operators | 3.81 (0.14) | 3.96 |
| 422 | Client Information Workers | 3.80 (0.15) | 3.95 |
| 814 | Rubber, Plastic and Paper Products Machine Operators | 3.80 (0.15) | 3.96 |
| 732 | Printing Trades Workers | 3.79 (0.14) | 3.94 |
| 731 | Handicraft Workers | 3.79 (0.14) | 3.93 |
| 516 | Other Personal Services Workers | 3.78 (0.14) | 3.92 |
| 742 | Electronics and Telecommunications Installers and Repairers | 3.76 (0.16) | 3.92 |
| 834 | Mobile Plant Operators | 3.76 (0.14) | 3.90 |
| 962 | Other Elementary Workers | 3.75 (0.16) | 3.91 |
| 835 | Ships' Deck Crews and Related Workers | 3.72 (0.14) | 3.86 |
| 221 | Medical Doctors | 3.72 (0.09) | 3.81 |
| 833 | Heavy Truck and Bus Drivers | 3.69 (0.11) | 3.80 |
| 541 | Protective Services Workers | 3.69 (0.14) | 3.82 |
| 441 | Other Clerical Support Workers | 3.67 (0.12) | 3.79 |
| 031 | Armed Forces Occupations, Other Ranks | 3.62 (0.09) | 3.71 |
| 831 | Locomotive Engine Drivers and Related Workers | 3.48 (0.15) | 3.62 |
| 816 | Food and Related Products Machine Operators | 3.47 (0.14) | 3.61 |

## Appendix 2. Supplementary findings

**Table S2.** Baseline characteristics of young employees entering the labor market across. The Danish Work Life Course Cohort study, Denmark, 2010–2019.

|  |  | Knowledge | Service | Care | Education | Industrial | Construction | Other |
| --- | --- | --- | --- | --- | --- | --- | --- | --- |
|  | Total (n) | 25,354 | 184,996 | 25,876 | 17,438 | 20,903 | 6,340 | 20,278 |
|  |  |  |  |  |  |  |  |  |
| Sex | |  |  |  |  |  |  |  |
|  | Women | 45.1% | 54.5% | 71.9% | 47.6% | 42.7% | 12.4% | 55.6% |
|  | Men | 54.9% | 45.5% | 28.1% | 52.4% | 57.3% | 87.6% | 44.4% |
| Age in years, Mean (SD) | | 21.9 (4.1) | 19.3 (3.6) | 20.6 (3.5) | 22.7 (4.3) | 21.4 (4.5) | 21.1 (3.5) | 21.2 (3.6) |
|  | 15-19 | 33.1% | 64.9% | 43.6% | 28.7% | 42.7% | 37.6% | 43.5% |
|  | 20-25 | 46.5% | 27.3% | 45.1% | 40.6% | 36.5% | 51.1% | 44.0% |
|  | >25 | 20.4% | 7.8% | 11.2% | 30.8% | 20.9% | 11.3% | 12.5% |
| Civil status | |  |  |  |  |  |  |  |
|  | Single or living alone | 30.8% | 27.1% | 34.9% | 30.6% | 24.6% | 27.6% | 31.3% |
|  | Married or registered partner | 52.9% | 64.2% | 59.5% | 45.4% | 54.2% | 61.5% | 62.0% |
|  | Unknown | 16.3% | 8.7% | 5.6% | 24.0% | 21.2% | 10.9% | 6.7% |
| Ethnicity | |  |  |  |  |  |  |  |
|  | Born in Denmark and no migration background | 67.6% | 76.7% | 77.1% | 56.6% | 65.2% | 82.1% | 77.8% |
|  | Born outside Denmark | 27.2% | 15.7% | 16.3% | 39.8% | 32.1% | 15.5% | 15.7% |
|  | Born in Denmark and both parents are immigrants | 5.2% | 7.6% | 6.6% | 3.7% | 2.8% | 2.4% | 7.5% |
| Annual disposable income (EUR). Mean (SD) | | 12,826  (11,242) | 7,759 (7,102) | 9,787 (6,638) | 12,057 (9,657) | 13,408 (12,837) | 15,834 (10,432) | 6,154 (6,532) |
|  | Mean income EUR (SD) |  |  |  |  |  |  |  |
|  | < 10,000 EUR | 51.1% | 73.2% | 58.1% | 52.1% | 47.4% | 32.3% | 84.4% |
|  | 10,000 – 20,000 EUR | 30.9% | 21.0% | 35.1% | 29.9% | 30.7% | 40.1% | 12.1% |
|  | 20,000 – 30,000 EUR | 9.9% | 4.5% | 5.1% | 10.9% | 13.7% | 18.0% | 2.5% |
|  | 30,000 – 40,000 EUR | 5.1% | 0.9% | 1.3% | 6.1% | 5.6% | 7.6% | 0.1% |
|  | > 40,000 EUR | 3.0% | 0.4% | 0.4% | 1.1% | 2.5% | 2.0% | 0.1% |
| Employment sector | |  |  |  |  |  |  |  |
|  | Public | 38.4% | 28.1% | 91.3% | 86.8% | 22.7% | 18.0% | 56.9% |
|  | Private | 61.6% | 71.9% | 8.7% | 13.2% | 77.3% | 82.0% | 43.1% |
| Occupational level | |  |  |  |  |  |  |  |
|  | Professional | 23.5% | 3.2% | 12.7% | 58.4% | 10.6% | 3.0% | 0.3% |
|  | Semi-professional and clerical | 43.3% | 63.2% | 73.2% | 26.9% | 30.6% | 10.3% | 1.5% |
|  | Routine | 33.1% | 33.6% | 14.1% | 14.6% | 58.7% | 86.6% | 98.2% |
| Somatic diseases | |  |  |  |  |  |  |  |
|  | No | 94.3% | 93.0% | 92.9% | 94.8% | 94.3% | 91.2% | 92.5% |
|  | Yes | 5.7% | 7.0% | 7.1% | 5.2% | 5.7% | 8.8% | 7.5% |
| Mental disorders | |  |  |  |  |  |  |  |
|  | No | 93.9% | 93.3% | 88.7% | 93.9% | 93.5% | 90.0% | 91.0% |
|  | Yes | 6.1% | 6.7% | 11.3% | 6.1% | 6.5% | 10.0% | 9.0% |
| Annual health service use, Mean (SD) | | 11.3  (14.4) | 11.0 (13.6) | 13.8 (16.8) | 11.0  (13.7) | 10.4  (13.7) | 9.4  (12.1) | 13.1 (15.9) |
|  | 0 | 26.9% | 23.1% | 15.7% | 37.6% | 31.3% | 22.8% | 14.1% |
|  | 1-3 | 19.6% | 21.2% | 18.2% | 17.4% | 19.6% | 24.1% | 20.6% |
|  | 4-7 | 19.3% | 19.9% | 19.7% | 16.6% | 18.4% | 21.4% | 20.0% |
|  | 8-15 | 18.5% | 19.4% | 22.1% | 15.2% | 17.4% | 19.3% | 21.8% |
|  | 16+ | 15.7% | 16.4% | 24.4% | 13.1% | 13.2% | 12.4% | 23.5% |
|  |  |  |  |  |  |  |  |  |
| Sickness absence | |  |  |  |  |  |  |  |
|  | Mean days (SD) | 3.2 (11.1) | 2.6 (10.2) | 7.4 (20.2) | 2.4 (12.0) | 3.8 (12.5) | 5.2 (14.0) | 3.2 (10.2) |

**Table S3.** Rate ratios (RR) and 95% confidence intervals (CI) for the stepwise adjustment for the association between one point increase in influence at work at occupational level and annual sickness absence days among 301,185 young Danish employees and across occupational group. The Danish Work Life Course Cohort study, Denmark, 2010–2019.

|  | Model 1 | Model 2 | Model 3 | Model 4 | Model 5 | Model 6 |
| --- | --- | --- | --- | --- | --- | --- |
|  | RR (95% CI) | RR (95% CI) | RR (95% CI) | RR (95% CI) | RR (95% CI) | RR (95% CI) |
| All | 0.32 (0.30-0.35) | 0.31 (0.29-0.34) | 0.40 (0.38-0.43) | 0.40 (0.38-0.43) | 0.54 (0.50-0.58) | 0.71 (0.66-0.77) |
| Knowledge | 0.51 (0.42-0.62) | 0.43 (0.35-0.53) | 0.57 (0.46-0.70) | 0.56 (0.45-0.68) | 0.70 (0.57-0.85) | 0.71 (0.56-0.90) |
| Service | 0.56 (0.49-0.64) | 0.56 (0.49-0.63) | 0.69 (0.60-0.78) | 0.69 (0.60-0.78) | 0.76 (0.67-0.87) | 0.88 (0.77-1.02) |
| Care | 0.57 (0.48-0.67) | 0.48 (0.41-0.56) | 0.54 (0.45-0.65) | 0.54 (0.45-0.65) | 0.57 (0.47-0.69) | 0.62 (0.52-0.75) |
| Education | 0.11 (0.08-0.17) | 0.16 (0.11-0.24) | 0.13 (0.08-0.20) | 0.14 (0.09-0.38) | 0.35 (0.23-0.55) | 0.57 (0.37-0.89) |
| Industrial | 0.36 (0.31-0.41) | 0.33 (0.29-0.38) | 0.42 (0.36-0.49) | 0.42 (0.36-0.49) | 0.49 (0.43-0.56) | 0.84 (0.70-1.01) |
| Construction | 0.93 (0.41-2.15) | 0.93 (0.41-2.08) | 0.89 (0.40-1.98) | 0.96 (0.42-2.22) | 0.91 (0.43-1.95) | 1.49 (0.63-3.52) |
| Other | 0.16 (0.08-0.31) | 0.14 (0.07-0.30) | 0.28 (0.14-0.59) | 0.24 (0.11-0.50) | 0.60 (0.28-1.31) | 1.30 (0.46-3.69) |

*Model 1: Adjusted rate ratio for sex, age, years since labor market entry, years with employment, and calendar year.
Model 2: Further adjusted for civil status, and ethnicity.
Model 3: Further adjustment for income, employment sector, occupational grade, and childhood socioeconomic status.
Model 4: Further adjustment for health service use, and somatic diseases and mental disorder before labor market entry.
Model 5: Further previous sickness absence.
Model 6: Further adjusted for physical workload (fully adjusted model)*

**Table S4.** Predicted reduction in sickness absence days with a simulated increase in influence at work with one standard deviation based on fully adjusted rate ratios among 301,185 young Danish employees and across occupational group. The Danish Work Life Course Cohort study, Denmark, 2010–2019.

|  |  | Predicted change in sickness absence days | | |
| --- | --- | --- | --- | --- |
|  | Average  simulated  increase  in influence (percentage increase) | Average individual-level change (95% CI) | Total population-level change (95% CI) | Percentage population-level change (95% CI) |
| All | 0.09 (2.3%) | -0.16 (-0.19; -0.13) | -126,400 (-146,914; -105,885) | -2.9 (-3.3; -2.4) |
| Knowledge | 0.11 (2.7%) | -0.19 (-0.30; -0.09) | -14,879 (-22,980; -6,778) | -3.3 (-5.1; -1.5) |
| Service | 0.09 (2.4%) | -0.04 (-0.09; +0.002) | -17,308 (-35,410; +794) | -1.1 (-2.2; +0.1) |
| Care | 0.08 (2.0%) | -0.37 (-0.49; -0.24) | -54,195 (-73,016; -35,375) | -3.5 (-4.7; -2.3) |
| Education | 0.08 (1.9%) | -0.18 (-0.26; -0.10) | -10,585 (-15,153; -6,018) | -4.2 (-6.0; -2.4) |
| Industrial | 0.11 (2.9%) | -0.12 (-0.24; +0.01) | -6,229 (-12,996; +538) | -2.1 (-4.4; +0.2) |
| Construction | 0.10 (2.5%) | +0.29 (-0,03; +0.60) | +3,942 (-392; +8276) | +4.3 (-0.4; +9.0) |
| Other | 0.04 (1.1%) | +0.05 (-0.14; +0.22) | +1,265 (-4,291; +6,820) | +1.2 (-3.9; +6.2) |

**Table S5.** Predicted reduction in sickness absence days with a simulated increase in influence at work with one standard deviation based on fully adjusted rate ratios (RR) among 160,104 women and 141,081 men and across occupational group. The Danish Work Life Course Cohort study, Denmark, 2010–2019.

|  |  | Predicted change in sickness absence days | |
| --- | --- | --- | --- |
|  | Fully adjusted  RR (95% CI) | Average individual-level change  (95% CI) | Total population-level change  (95% CI) |
| Women |  |  |  |
| All | 0.62 (0.57-0.67) | -0.24 (-0.28; -0.20) | -105,237 (-124,078; -86,396) |
| Knowledge | 0.72 (0.57-0.91) | -0.22 (-0.36; -0.08) | -7,190 (-11,741; -2,639) |
| Service | 0.83 (0.72-0.96) | -0.07 (-0.13; -0.02) | -15,715 (-27,938; -3,493) |
| Care | 0.83 (0.69-1.00) | -0.15 (-0.29; -0,01) | -16,750 (-32,909; -591) |
| Education | - | - | - |
| Industrial | 0.66 (0.44-0.99) | -0.24 (-0.45; -0.03) | -4,833 (-9,125; -540) |
| Construction | 0.28 (0.08-1.00) | -0.62 (-1.25; 0.002) | -950 (-1,903; 2) |
| Other | 1.13 (0.40-3.22) | 0.02 (-0.17; 0.22) | 419 (-3,013; 3,851) |
|  |  |  |  |
| Men |  |  |  |
| All | 0.61 (0.57-0.65) | -0.22 (-0.25; -0.19) | -77,185 (-86,838; -67,533) |
| Knowledge | 0.64 (0.55-0.76) | -0.23 (-0.31; -0.14) | -10,040 (-13,699; -6,381) |
| Service | 0.79 (0.72-0.86) | -0.09 (-0.11; -0.06) | -15,922 (-21,622; -10,222) |
| Care | 0.42 (0.33-0.54) | -0.67 (-0.84; -0.51) | -23,311 (-29,103; -17,519) |
| Education | 0.36 (0.22-0.51) | -0.24 (-0.36; -0.13) | -7,381 (-10,923; -3,839) |
| Industrial | 0.92 (0.79-1.07) | -0.06 (-0.15; 0.03) | -1,905 (-4,848; 1,037) |
| Construction | 1.74 (1.05-2.87) | 0.39 (0.06; 0.71) | 4,737 (782; 8,691) |
| Other | - | - | - |

*Fully adjusted rate ratio for age, years since labor market entry, years with employment, and calendar year, civil status, ethnicity, income, employment sector, occupational grade, health service use, somatic diseases and mental disorder before labor market entry, physical workload, childhood socioeconomic status, and previous sickness absence.*

**Figure S1.** Distribution of occupational group since labor market entry among 160,104 women and 141,081 men 2010 and 2018. The Danish Work Life Course Cohort study, Denmark, 2010–2019.


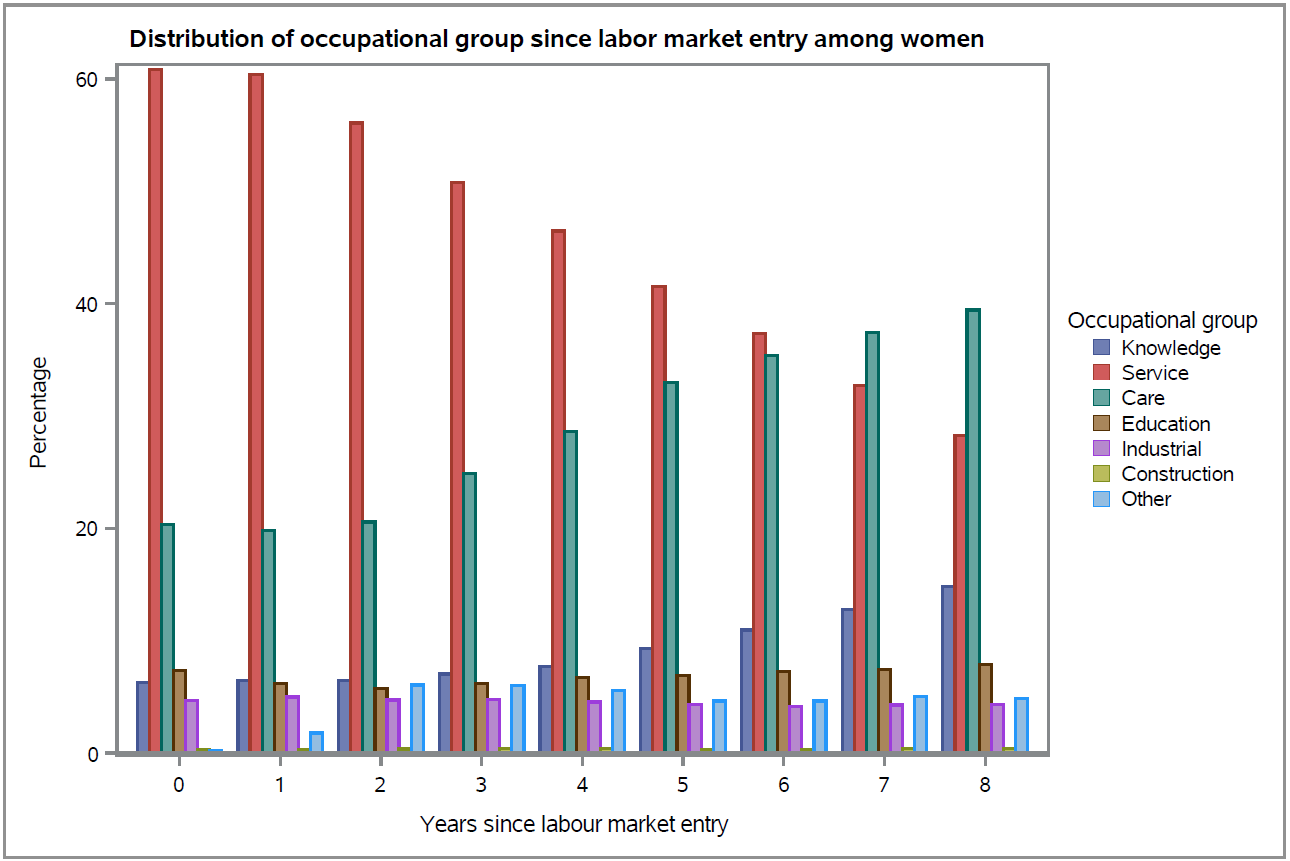


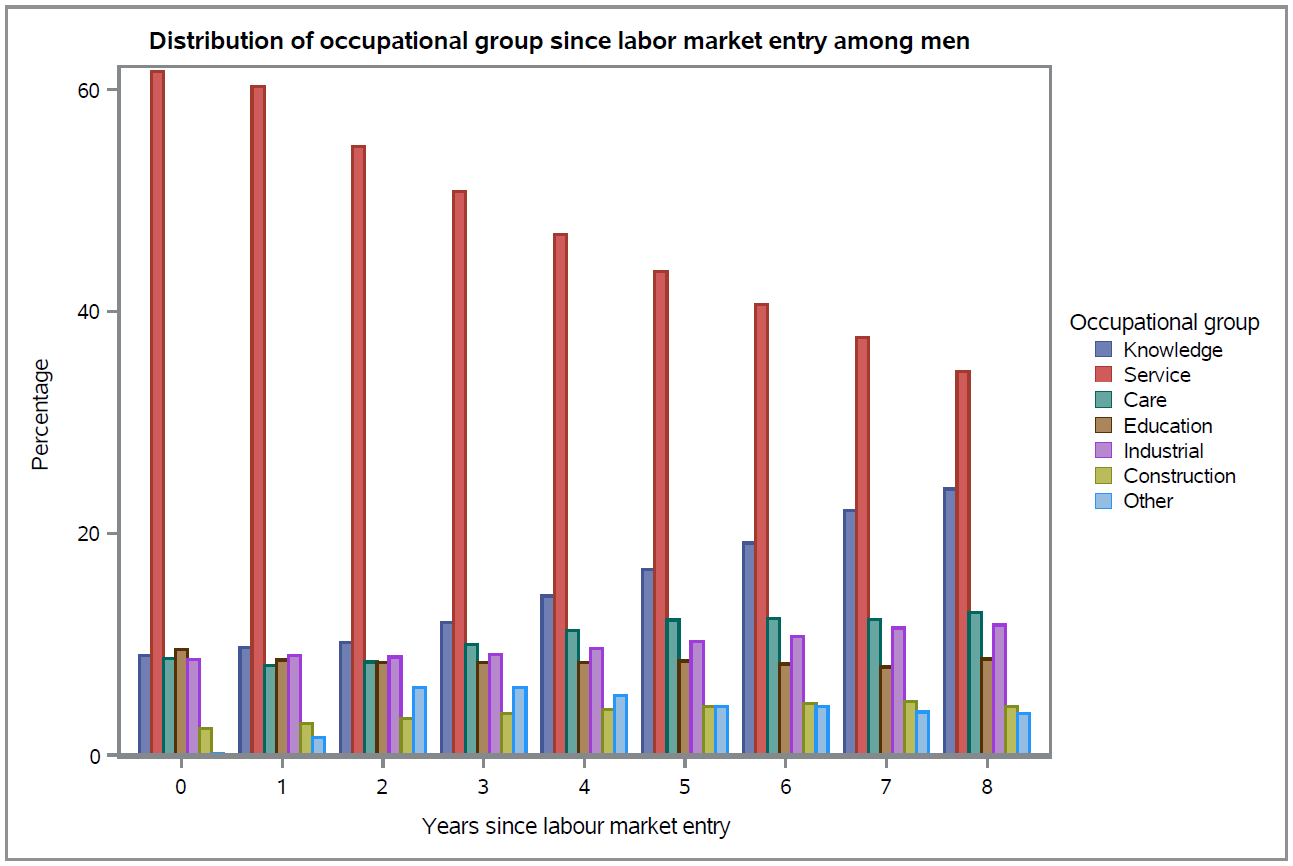

Supplement: Supplementary file 1 [file Supplementaryfile1.docx]
